# Minimally Invasive Technique in the Management of Tibial Pilon Fractures: New Approach and Promising Results

**DOI:** 10.1155/2023/1272490

**Published:** 2023-03-21

**Authors:** Yassine Ben Bouzid, Rida-Allah Bassir, Monsef Boufettal, Jalal Mekkaoui, Mohamed Kharmaz, Moulay Omar Lamrani, Mohamed Saleh Berrada

**Affiliations:** Department of Orthopedic and Trauma Surgery, Ibn Sina University Hospital, Rabat, Morocco

## Abstract

**Background:**

Comminuted tibial pilon fractures are induced by high-energy mechanisms and are often associated with soft tissue injuries. Their surgical approach is problematic due to postoperative complications. Minimally invasive management of these fractures has a considerable advantage in preserving the soft tissue and the fracture hematoma.

**Materials and Methods:**

We conducted a retrospective study of a series of 28 cases treated at the Orthopedic and Traumatological Surgery Department of the CHU Ibn Sina in Rabat over a period of 3 years and 9 months, from January 2018 to September 2022.

**Results:**

After a mean follow-up of 16 months, 26 cases had good clinical results according to the Biga SOFCOT criteria and 24 cases had good radiological results according to the Ovadia and Beals criteria. No cases of osteoarthritis were observed. No skin complications were reported.

**Conclusion:**

This study highlights a new approach that deserves to be considered for this type of fracture as long as no consensus has been given.

## 1. Introduction

First described by Hulscher [1] in 1911, the tibial pilon represents the distal end of the tibia and extends from the tibiotalar joint up to 8 cm distal to this articular surface [2]. Tibial pilon fractures account for 5–7% of all tibial fractures [3–5] and most often occur as a result of high-energy trauma. These injuries most often produce significant comminution with fragment displacement and severe soft tissue trauma [6] in a young population with high functional demand. The fibula is also fractured in 85% of high-energy tibial pilon fractures [7]. These fractures can also occur in an elderly population following low-energy trauma due to deterioration of bone quality. In this case, the soft tissues are compromised by comorbidities such as diabetes, arteriopathy, or medication.

The treatment of these fractures is still the subject of research [8, 9] due to the lack of consensus. Indeed, there is a multitude of therapeutic strategies, implants, and approaches that can be used with different results [10–17].

While the surgical treatment must take into account the osseous component for stabilization, the importance of the soft tissues should not be neglected as they are subject to numerous skin and infectious complications [18]. The soft tissues surrounding the distal tibia are often compromised, which is crucial for preoperative planning including the timing of the surgery [19].

Complications are common, involving infection, compartment syndrome, and osteoarthritis [20]. Conditioned media from human osteoarthritic synovium induce inflammation in a synoviocyte cell line [21]. The main objective of surgical treatment is to avoid skin and infectious complications by meticulous preoperative planning and to avoid the occurrence of ankle osteoarthritis, which could compromise the functional prognosis in a young population [22].

Since there are not enough studies on this new approach for the treatment of comminuted pilon fractures, we will try to highlight this technique based on a series of 28 cases and, through the results, to assess the risk of skin complications and infection, the occurrence of osteoarthritis, and the functional prognosis of the patients. This work could also serve as a pilot study before conducting comparative studies.

## 2. Materials and Methods

### 2.1. Study Group

The present work is a retrospective study of 28 cases of comminuted distal tibia fractures treated by minimally invasive plate osteosynthesis (MIPO) over a period from January 2018 to September 2022, collected in the Department of Orthopedic and Trauma Surgery at Ibn Sina University Hospital in Rabat.

Inclusion criteria were as follows: (1) age > 18 years; (2) patients admitted for tibial pilon fracture isolated or associated with other fractures; (3) patients treated for comminuted tibial pilon fracture; and (4) patients presenting with or without skin suffering.

We excluded from our study open fractures, patients who had received another therapeutic option, patients who were lost to follow-up, and incomplete records.

Gender, comorbidities, mechanism of injury, affected side, and laterality were not included in the selection criteria.

The study of the files was based on exploitation of the patients' medical files, the operating reports, and the consultation registers. Data were entered on an Excel spreadsheet.

Skin lesions were evaluated according to the Tscherne classification. The anatomopathological aspects of the fractures were studied on the basis of a descriptive radiological analysis using the AO/OTA classification system.

The clinical assessment focused first on the skin condition, then on the presence of pain, and the assessment of joint mobility. Radiographic control assessed the healing status of the bones.

### 2.2. Description of the Technique

**Figure 1:**
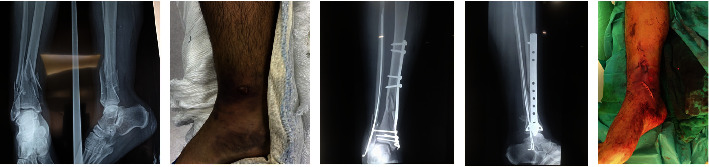
Pre- and postoperative clinical and radiographic images of a 33-year-old patient treated with MIPO.

**Figure 2:**
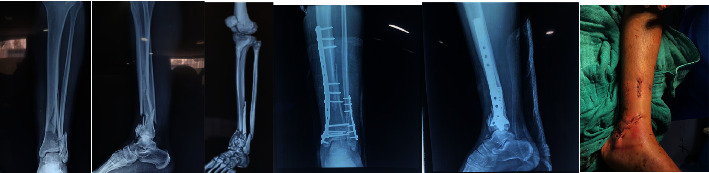
Pre- and postoperative radiographic and clinic check-up of a 42-year-old patient operated on for a 43-C3 fracture using a minimally invasive approach.

The first step in tibial reduction is fixation of the lateral malleolus. Depending on the condition of the skin around the fibula, the fibular fracture will be stabilized by percutaneous pinning (Figure 1) or by open reduction and internal fixation (Figure 2). Tibial comminution limits the use of reduction forceps. After stabilization of the lateral malleolus, residual tibial displacement is observed. Tibial pilon fractures are most often displaced in varus and recurvatum. The first step is reduction of the articular surface, which must be anatomical. Then, a transcalcaneal Steinmann pin is placed which allows traction and correction of the problems in the frontal plane. The recurvatum is corrected with a towel roll placed under the leg in regard to the fracture site. The incision is oblique to the medial malleolus and about 5 cm long. After preparing the tunnel with the rugine, the plate is inserted subcutaneously. Using the C-arm, the reduction and length of the plate are evaluated. Any displacement in the sagittal plane must be corrected. The residual varus or valgus displacement will be eliminated by the effect of the plate. The first screw placed is the one closest to the fracture site on the distal fragment. This screw is bicortical and allows the entire distal fragment to be recalled and attached to the plate. Next, the proximal screws are used to stabilize the proximal fragment. They can be unicortical or bicortical if a locked plate is used. If a conventional plate is used, all screws must be bicortical. Finally, the screwing of the other holes will be completed.

### 2.3. Postoperative Management

All our patients benefited from medical treatment for pain, thromboprophylaxis for 14 days and antibiotic prophylaxis for 48 hours. Immobilization was systematically performed for 6 weeks.

The day after surgery, a lymphatic drainage massage is started with knee mobilization and isometric contractions of the leg muscles.

Two orthogonal radiographic views were performed immediately after surgery (Figures 1 and 2) and at each follow-up.

## 3. Results

The average age of our patients was 41.5 years (between 28 and 50 years), with a sex ratio of 1.3 (16 males and 12 females). The trauma was of high energy in all our patients. The fracture occurred after a road traffic accident in 20 cases and due to a fall from great height in 8 cases. The right side was affected in 17 cases and the left in 11 cases. The patients presented to the emergency room on average within two hours of the trauma and were operated on within 4 to 24 hours of the trauma.

The Tscherne classification was used to evaluate skin injuries. 6 cases had a grade I clinical appearance; 18 cases had grade II skin contusion; only two cases presented with simple ankle edema (Table 1). A complete radiological workup was performed, including standard radiographs with two views (anteroposterior and lateral), with additional CT scans. The Ruedi and Allgower classification was not applied since it does not consider the metaphyseal segment of the tibia nor the malleolar component. Our patients were classified according to the AO model. 7 patients had a type A2 lesion; 11 cases had a type A3 lesion; 6 patients had a type C2 lesion; and 4 patients had a type C3 lesion. The fracture of the lateral malleolus was present in all patients (Table 2). No dislocation was noted. The neurovascular system was intact in all our patients.

**Figure 3:**
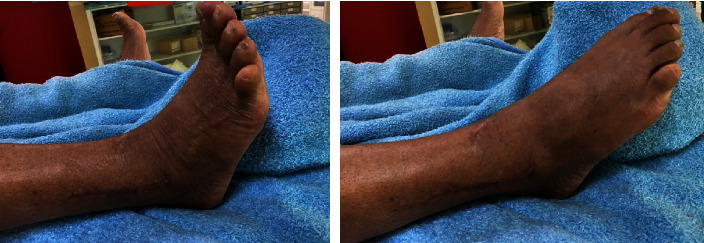
Clinical control of a patient with full recovery of plantar flexion and dorsal flexion of the ankle.

The average follow-up was 16 months. Monthly control was recommended for the first 6 months, followed by long-term control every 6 months. Clinical evaluation was based on the Biga SOFCOT criteria [23] which take into consideration pain, walk, mobility, and edema (Table 3). Radiological control assessed the quality of reduction, consolidation, joint congruence, and the presence of signs of osteoarthritis. The results were evaluated according to the Ovadia and Beals criteria [24] (Table 4). Bone healing was obtained on average after 5 months of surgery. No skin complications were noted; joint mobility was symmetrical to the healthy side (Figure 3); intermittent pain was reported in 2 cases. No cases of arthritis were observed.

## 4. Discussion

Tibial pilon fractures represent a surgical challenge, and their management remains difficult because they are intra-articular lesions associated with various degrees of soft tissue injury. The mechanism is variable and most often violent, resulting in significant soft tissue damage and highly comminuted displaced fractures [25]. There are several classification systems, the most inclusive of which is the AO/OTA classification of tibial pilon fractures: extra-articular (43-A); partial articular (43-B); and complete articular (43-C). These types are subdivided according to comminution. Soft tissue injuries are classified according to the Tscherne classification.

The goal of surgical treatment is restoration of limb length and alignment, as well as anatomic reduction of the articular surface [2]. Although several techniques have been described for the management of pilon fractures, few have addressed comminuted distal tibial fractures. Minimally invasive osteosynthesis was designed to minimize the risk of infection and pseudarthrosis. It also allows the preservation of the biological arsenal during bone healing by conserving the fracture hematoma and avoiding periosteal stripping [26]. Kim et al. [27] studied type C fractures treated with MIPO with satisfactory results and significantly fewer skin complications. Baumgaertel et al. [28] reported callus formation in the first weeks following biological fixation. The onset of bone healing was observed after 5 to 6 weeks following open anatomical reduction. Davidovitch et al. [29] conducted a comparative study between 2-stage internal osteosynthesis and external fixation. There was no significant difference between both groups in regaining joint range of motion; postoperative skin complications were significantly less for ORIF. A study was conducted by Biz et al. [30] evaluating the medium- and long-term results in 94 patients with 43-B and 43-C fractures using all three techniques: ORIF, MIPO, and external fixation. The radiographic and clinical results when the MIPO technique was used were slightly better than those of ORIF and much better than those of external fixation.

In our group of patients, 28 cases of comminuted tibial pilon fractures were operated on using the MIPO technique. The clinical evaluation showed good results in 92.86% of the cases and good radiological results in 85.71% of the cases, which are better than the results of other techniques reported in the literature. Consolidation was obtained after 4 months, and no cases of arthrosis or skin complications were reported. On the other hand, no case of a 43-B fracture was operated on using this technique, despite the fact that this type of fracture was not an excluding criterion. The limited sample size is also a limitation of this study.

## 5. Conclusion

In conclusion, based on the results of our study, the MIPO technique should be of considerable interest in the management of tibial pilon fractures, particularly when the soft tissue is compromised and exposure of the fracture site would be fatal. This technique, through very short incisions, provides soft tissue protection. This approach avoids manipulation and devascularization of the fragments and preserves the fractured hematoma, thus allowing for adequate biological consolidation.

## Figures and Tables

**Table 1:** Tscherne classification for soft tissue injuries.

Tscherne grade	Number of cases	Percentage
Grade 0	4	14.29
Grade I	6	21.43
Grade II	18	64.29
Grade III	0	0

**Table 2:** Distribution of cases by fracture type according to the AO/OTA classification system.

AO/OTA classification	A			B			C		
	A1	A2	A3	B1	B2	B3	C1	C2	C3
Number of cases	0	7	11	0	0	0	0	6	4
Percentage	0	25	39.29	0	0	0	0	21.43	14.29

**Table 3:** Clinical evaluation according to the Biga SOFCOT criteria [23].

Results	Number of cases	Percentage
Good	26	92.86
Acceptable	2	7.14
Insufficient	0	0
Poor	0	0

**Table 4:** Control of the radiological quality of reduction according to the Arlettaz criteria [24].

Quality of radiological reduction	Number of cases	Percentage
Good	24	85.71
Fair	4	14.29
Poor	0	0

## Data Availability

The datasets used and analysed during the study are available from the corresponding author.

## Abbreviations

AO/OTA: AO Foundation/Orthopaedic Trauma Association
MIPO: Minimally invasive percutaneous osteosynthesis
ORIF: Open reduction and internal fixation.
